# Wound healing in immunocompromised dogs: A comparison between the healing effects of moist exposed burn ointment and honey

**DOI:** 10.14202/vetworld.2020.2793-2797

**Published:** 2020-12-29

**Authors:** Musa Alshehabat, Wael Hananeh, Zuhair Bani Ismail, Safwan Abu Rmilah, Mahmoud Abu Abeeleh

**Affiliations:** 1 Department of Veterinary Clinical Sciences, Faculty of Veterinary Medicine, Jordan University of Science and Technology, Irbid 22110, Jordan; 2 Department of Pathology and Public Health, Faculty of Veterinary Medicine, Jordan University of Science and Technology, Irbid 22110, Jordan; 3 Department of Surgery, Faculty of Medicine, University of Jordan, Amman, 11942 Jordan

**Keywords:** epithelialization, open wound healing, wound contraction, wound dressings

## Abstract

**Background and Aim:**

Natural topical products have been used to enhance wound healing, especially in immunocompromised animals. The aims of this study were to evaluate and to compare the effects of moist exposed burn ointment (MEBO) and honey on the healing of full-thickness skin wounds in immunocompromised dogs.

**Materials and Methods:**

The study was conducted using 12 adults, apparently healthy mongrel dogs. Immunosuppression was induced in six dogs by oral administration of prednisone (2 mg/kg) and azathioprine (2 mg/kg), once a day for 21 days. On each dog, a total of 9, 1.5 cm in diameter full-thickness skin circular wounds were created aseptically in the thoracolumbar area under general anesthesia using customized skin punch biopsy kit. In a random fashion, three wounds in each dog were treatment using MEBO (contains b-sitosterol, baicalin, and berberine as active ingredients in a base of beeswax and sesame oil), honey or no treatment (control), once per day for 21 days. Wounds were grossly evaluated once a day for signs of inflammation or infection. In addition, biopsy specimens and digital imaging data of each wound were obtained on days 7, 14, and 21 for histopathological evaluation of the healing process.

**Results:**

Wounds in immunocompromised dogs appeared to heal significantly in a slower fashion than in non-immunocompromised counterparts. Digital analysis data showed that MEBO-treated wounds expressed better epithelialization area, faster contraction, and smaller wound area percentage when compared with honey-treated wounds. Histopathological analysis showed significantly higher angiogenesis scores in MEBO-treated wounds when compared with other treatments.

**Conclusion:**

Results of this study showed that MEBO resulted in significant enhancement of wound healing in both healthy and immunocompromised dogs. However, when compared to honey, the wound healing effect of MEBO was superior to that of honey.

## Introduction

While the principles of standard wound care are similar to all wounds, there are several reported comorbidities that influence the healing of cutaneous wounds, including diabetes mellitus, nutritional status, and medications corticosteroids [1-3]. In fact, corticosteroids have been reported to impair wound healing at different stages of the healing cascade [4,5]. Corticosteroids are commonly used in small animal practice to manage various diseases, including allergic disorders, inflammation, immune-mediated diseases, hypoadrenocorticism, and spinal injuries [3]. Clinically, the duration of corticosteroid treatment varies with the etiology and response to treatment and potentially the development of undesired side effects [6]. One of the most important side effects of long-term corticosteroids treatment is immunosuppression [6,7].

Available literature in human medicine suggests that moist exposed burn ointment (MEBO) enhances wound healing, produces local analgesic effects, and reported no side effects [8-12]. Honey, on-the-other hand, is a natural product that has been used in the management of acute wounds, chronic wounds, and burn wounds in humans and animals [13,14]. Honey has been shown to possess many medicinal properties, including anti-inflammatory effects, analgesic effects, deodorizing effects, and hygroscopic effects. Clinically, honey has been shown to decrease wound exudation, enhance immune response at the wound site, stimulate healthy granulation tissue formation, minimize scar tissue formation, and reduce the incidence of wound infections [15-20].

The aims of this study were to evaluate and to compare the effects of MEBO and honey on the healing of full-thickness skin wounds in immunocompromised dogs.

## Materials and Methods

### Ethical approval

The study protocol was reviewed and approved by the Institutional Animal Care and Use Committee (IACUC) of Jordan University of Science and Technology (Grant No. 203-2016).

### Study location and period

This study was performed at the Laboratory Animal House of Jordan University of Science and Technology from January to March 2016.

### Animals

A total of 12 healthy, adult mongrel dogs, ranging in age from 1 to 5 years, were obtained from a local breeder for the purpose of this study. The mean body weight of the dogs was 20 kg. Dogs were subjected to complete physical examination, including complete blood cell count and serum biochemistry analyses to evaluate the general health status of the animals before the inclusion in the study. Dogs with history or clinical signs suggestive of internal organ dysfunction or skin disease were excluded from the study. Selected dogs were vaccinated and dewormed at the time of admission to the hosting facility. Each dog was identified using collar ID and housed separately in cages and was provided with daily dry food (Benson; Adfico, Jordan) and *ad libitum* source of clean water.

### Study design and wound creation

Selected dogs were randomly allocated to control (n=6) and immunocompromised (n=6) group. In the immunocompromised group, immunosuppression was achieved by the administration of prednisone (5 mg/tablet; Douglas Pharmaceuticals, USA) at a dose of 2 mg/kg and azathioprine (50 mg/tablet; Bausch Health Inc., Canada) at a dose of 2 mg/kg orally once per day for 21 days. In the control group, dogs were not administered any medications during this period.

Wounds were created on the back of each dog under strict aseptic conditions and under general anesthesia using intramuscular injection of Ketamine (Ketamine, Alfasan, Woerden, Holland) at a dose of 10 mg/kg and Xylazine (Xyla-Ject, Adwia, Egypt) at a dose of 0.5 mg/kg. In each of the dogs, a total of nine, 1.5 cm in diameter circular wounds were created in the thoracolumbar area using customized circular dermal template. Wounds were placed 2-3 cm apart from each other. Wounds were covered with sterile stressing only for the first 24 h. During treatment, wounds were exposed to air and inspected daily for signs of inflammation or infection until complete healing.

### Wound treatment

Wounds in each row were randomly allocated to receive once-per-day treatment of the following products: Locally produced honey, MEBO (contains b-sitosterol, baicalin, and berberine as active ingredients in a base of beeswax and sesame oil; Julphar, Gulf Pharmaceutical Industrial Co., United Arab Emirates) or no treatment for 21 days or until the wounds are completely healed. Treatment products were applied gently to fill the wound area. Wounds were gently flushed each day with normal saline to eliminate debris and crusts before applying treatments. Wounds were allowed to heal by second intention without applying any bandages. Elizabethan collar was applied to each dog throughout the study.

### Gross evaluation of the wounds

Wounds were grossly evaluated on a daily basis throughout the duration of the study for signs of inflammation or infection. The evaluation was performed at the time of treatment application in the morning for the following signs: Hyperemia, edema, and exudate or pus discharge.

### Digital data analysis

Images of the wounds were obtained using a digital camera on days 7, 14, and 21. A standard reference metal ruler was placed adjacent to the wound before photographs were obtained to account for magnification error. Images were then downloaded and analyzed using image analysis software (ImageJ, National Institute of Health, USA) [21]. The following parameters were obtained: Unhealed wound area (cm^2^), epithelialization area (cm^2^), wound area percentage, and wound contraction percentage. The epithelialization area (cm^2^) was calculated by subtracting the measured unhealed wound area of a given day from the initial unhealed area of the same wound [21], and the percentage of wound area was calculated for each wound using the formula (Wound area [%] = 100 [wound area on day *x*/initial wound area]), while wound contraction rate was calculated using the formula (wound contraction rate = 100 [initial wound area – area of the given wound at evaluation time]/initial wound area).

### Histopathological evaluation

Tissue biopsies were obtained from the wounds once per week using a circular excisional biopsy. Biopsy samples were obtained under general anesthesia using propofol (Diprivan, 10 mg/ml; Fresenius, USA) at a dose of 6 mg/kg, intravenously. Wounds in first row were biopsied at week 1; wounds in the second row were biopsied at week 2; and wounds in the third row were biopsied at week 3. The obtained tissue samples were fixed in 10% formalin and processed for routine histopathological examination. A 5 μm thick sections of tissues were made and stained by Hematoxylin and Eosin. The stained sections were blindly examined by board-certified pathologist. A scoring system was developed to evaluate different histopathological parameters (Table-1) [13].

**Table-1:** Histopathological scoring system used to evaluate healing of skin wounds.

Histopathological parameters	Score
Re-epithelialization	
None	0
Partial	1
Complete but with immature epithelium	2
Complete with mature epithelium	3
Granulation tissue	
None or immature	0
Low amount	1
Moderate degree of maturation	2
Mature	3
Collagen accumulation	
None	0
Low amount	1
Moderate	2
High amount	3
Inflammatory cell	
None	0
Low amount	1
Moderate	2
High amount	3
Angiogenesis	
None	0
<5 veins	1
6-10 veins	2
More than 10 veins	3
Ulcer	
None	0
Very small	1
Large	2
Large or deep, abscess formation	3

### Statistical analysis

The data were presented as mean±standard deviation for the digital imaging parameters (unhealed wound area [cm^2^], epithelialization area [cm^2^], wound area percentage, and wound contraction percentage) in MEBO-treated wounds, honey-treated wounds, and untreated control wounds at each of the time points (days 7, 14, and 21). Student independent t-test was used to detect statistical differences in the evaluated parameters between groups at each time point. The Mann–Whitney test was used to detect statistical differences in the evaluated histological parameters (re-epithelialization, granulation, collagen accumulation, inflammatory cells, angiogenesis, and ulcer) between groups at each time point. Within groups, differences in the evaluated histological parameters between wounds were evaluated using non-parametric Friedman ANOVA test. *Post hoc* evaluation between treatments was performed using the Wilcoxon sign rank test. Differences were considered statistically significant at p<0.05. The analysis was conducted using statistical software (SPSS, Version 23.0, SPSS Inc, Chicago, USA).

## Results

### Gross evaluation

In general, wound healing occurred at a faster rate in healthy dogs as compared to immunocompromised counterparts. In the healthy dogs, signs of inflammation appeared to have subsided during the days 4-6 with no evidence of pus discharge in any of the evaluated wounds. In the immunocompromised group, signs of inflammation appeared to have subsided at the beginning of the 2^nd^ week with no evidence of pus discharge in any of the evaluated wounds. At the time of treatment application, the dried crusts or exudation that formed at each wound appeared to be easily cleaned off in the MEBO-treated wounds when compared to other treatments in both the control and immunocompromised groups.

### Digital imaging analysis

Table-2 shows the data obtained from the digital imaging analyses of wounds in healthy and immunocompromised groups. There was a significant difference (p<0.05) between unhealed wound area between honey-treated wounds and control wounds on day 7; unhealed wound area in honey-treated, MEBO-treated and control wounds on day 14; epithelization area between control wounds on day 21; wound area percentage in honey-treated, MEBO-treated and control wounds on day 14; and wound contraction percentage in honey-treated, and between MEBO-treated and control wounds on day 14. Within the healthy group, there were significant differences in the unhealed wound area on day 7, epithelization area on day 14 and day 21. Within the immunocompromised group, there were no significant differences in any of the evaluated parameters at any time point.

**Table-2:** Mean±SD values of digital imaging parameters of MEBO- and honey-treated skin wounds in healthy and immunocompromised dogs (n=12).

Parameters	Healthy dogs				Immunocompromised dogs		
	
	Time (days)	Honey	MEBO	Control	Honey	MEBO	Control
Unhealed wound area (cm^2^)	7	1.1±0.1†	1.4±0.2^HC^	1.2±0.1†	1.4±0.2	1.7±0.4	1.7±0.3
	14	0.1±0.0†	0.1±0.0†	0.1±0.1†	0.6±0.4	0.6±0.4	0.5±0.3
	21	0.0±0.0	0.0±0.0	0.0±0.0†	0.2±0.4	0.2±0.3	0.1±0.1
Epithelialization area (cm^2^)	7	0.0±0.0	0.0±0.0	0.0±0.0	0.0±0.0	0.0±0.0	0.0±0.0
	14	1.0±0.1^MC^	1.3±0.2^HC^	1.1±0.1^HM^	0.9±0.5	1.1±0.4	1.2±0.4
	21	1.1±0.1^M^	1.4±0.2^C^	1.2±0.1†	1.2±0.5	1.5±0.3	1.6±0.4
Wound area (%)	7	1.0±0.0	1.0±0.0	1.0±0.0	1.0±0.0	1.0±0.0	1.0±0.0
	14	0.1±0.0†	0.1±0.0†	0.1±0.1†	0.4±0.3	0.3±0.2	0.3±0.2
	21	0.0±0.0	0.0±0.0	0.0±0.0	0.2±0.3	0.1±0.1	0.1±0.1
Wound contraction (%)	7	0.0±0.0	0.0±0.0	0.0±0.0	0.0±0.0	0.0±0.0	0.0±0.0
	14	0.9±0.0†	0.9±0.0†	0.1±0.1†	0.6±0.3	0.7±0.2	0.7±0.2
	21	1.0±0.0	1.0±0.0	1.0±0.0	0.9±0.3	0.9±0.1	1.0±0.1

† The superscript sign Indicate significant differences between control and immunocompromised groups.

The superscript signs (H, C, and M) indicate significant difference within control or within immunocompromised group

### Histopathological evaluation

In general, wounds in both groups exhibited similar trends in healing. On day 7, the skin exhibited total loss of the entire epidermis (skin ulcer) with early granulation tissue formation, frequent new blood vessels (angiogenesis), scattered fibroblasts, and large numbers of neutrophils. There were significant differences in the median score of re-epithelialization of honey-treated wounds on day 14 and MEBO-treated wounds on day 21 between the control and the immunocompromised groups. Furthermore, there were significant differences in the median score of granulation tissue of untreated wounds between the control and the immunocompromised groups on day 14. Furthermore, there were significant differences in the median scores of inflammatory cells in MEBO-and untreated-wounds when compared to the control and immunocompromised groups on day 14. Within the control group, there were no significant differences in the median scores of the evaluated parameters in all wounds except angiogenesis on day 14. Within the immunocompromised group, there were no significant differences in the median scores in any of the evaluated parameters of all wounds. At the end of the experiment, wounds were completely closed with a continuous layer of stratified squamous epithelium. The dermis was devoid of adnexal structures and was mainly composed of dense collagenous connective tissue. The median scores of evaluated histopathological parameters are summarized in Table-3.

**Table-3:** Median score of various histopathological parameters of MEBO- and honey-treated wounds in healthy and immunocompromised dogs (n=12).

Parameter	Healthy dogs			

	Time (Days)	Honey	MEBO	Control
Re-epithelialization	7	0.50	0.00	0.50
	14	3.00*	3.00	3.00
	21	3.00	3.00*	3.00
Granulation tissue	7	3.00	3.00	2.00
	14	1.00	1.00	1.00
	21	1.00	1.00	1.00
Collagen accumulation	7	3.00	3.00	2.00
	14	3.00	3.00	3.00
	21	3.00	3.00	3.00
Inflammatory cells	7	3.00	3.00	2.50
	14	0.50	0.50*	0.00*
	21	0.00	1.00	0.00
Angiogenesis	7	3.00	3.00	3.00
	14	1.50	2.00	1.00
	21	1.00	1.00	1.00
Ulcer	7	3.00	3.00	2.50
	14	0.00	0.00	0.00
	21	0.00	0.00	0.00
Immunocompromised dogs				
Re-epithelialization	7	0.00	0.00	0.00
	14	1.00	0.00	1.00
	21	3.00	1.00	2.00
Granulation tissue	7	3.00	3.00	3.00
	14	2.00	2.00	2.00
	21	1.00	0.50	2.00
Collagen accumulation	7	3.00	2.00	2.00
	14	3.00	3.00	3.00
	21	3.00	1.50	2.00
Inflammatory cell	7	3.00	3.00	3.00
	14	2.00	2.00	1.50
	21	0.00	2.50	1.00
Angiogenesis	7	3.00	3.00	3.00
	14	1.50	2.00	2.00
	21	1.00	0.50	2.00
Ulcer	7	3.00	3.00	3.00
	14	2.50	3.00	2.00
	21	1.00	2.50	1.00

* Indicates significant differences in the evaluated parameters between control and immunocompromised groups (p<0.05)

## Discussion

Wound healing, especially in immunocompromised animals, presents challenging problems in clinical practice. Therefore, new and innovative topical wound medications are in constant quest. Ideally, topical wound treatment products must have a wide range of antibacterial properties while at the same time protect the wound environment from desiccation and other environmental factors and therefore enhancing the healing process [8-12]. In fact, most synthetic chemical products that are commonly used to manage open wounds may retard or interfere with the wound healing process [8-12]. In this regard, it is believed that natural products such as honey and MEBO may protect cutaneous wounds from infection and promote the healing process without causing any of the undesired effects caused by synthetic chemicals [8-12]. In this study, the wound healing properties of MEBO, a common herbal preparation of Chinese origin, and locally produced honey were evaluated in immunocompromised dogs. The topical application of MEBO has been reported previously to result in no adverse effects [22,23]. Similarly, in this study, all animals tolerated well the application of MEBO without showing any signs of local or systemic allergies or toxicities. As expected, topical application of MEBO and honey on wounds in immunocompromised dogs resulted in rapid resolution of wound inflammation with no evidence of pus discharge which was evident by the formation of thin crusts on the surface of treated wounds. In fact, previous research has reported that MEBO-treated wounds had softer eschar and were easier to debride when compared with honey-treated wounds [23]. On the contrary, the use of MEBO to treat wounds in donkeys was found inferior to pantheon gel [22]. Species differences (horses vs. dogs), wound location (dorsal thoracic vs. metacarpal wounds), and other environmental factors may account for the differences in response to various topical wound healing products in different animal species.

Analysis of digital images and histopathological scores of treated wounds using MEBO in this study has shown better wound healing as indicated by better epithelialization area, faster contraction, lower wound area percentage, and a significantly higher degree of angiogenesis when compared with honey-treated wounds and control wounds. These findings are similar to previously reported data [9-12]. It has been found that MEBO prevents bacteria from deeply penetrating wounded tissues and inhibits the proliferation of microorganisms laden in the wound bed [9-12]. Similar to previously reported findings, rapid re-epithelialization was observed in MEBO-treated animals [9-12].

## Conclusion

The results of this study showed that MEBO is safe and effective in promoting the healing of cutaneous wounds in healthy and corticosteroid-induced immunocompromised dogs. When compared to honey, the wound healing effects of MEBO were superior to those observed after the application of honey.

## Authors’ Contributions

MA designed the study and performed statistical analysis. WH performed histopathological evaluations. ZBI manuscript writing and data interpretation. SAR performed the experiment and collected data from the experimental subjects. MAA checked study design and performed scientific editing of the manuscript. All authors have read and approved the final manuscript.
